# Explaining the challenges of resources management and its underlying factors in COVID-19 era in Iran: a qualitative study

**DOI:** 10.1186/s12889-023-17045-0

**Published:** 2023-10-27

**Authors:** Vahid Vatandoost, Seyed Saeed Tabatabaee, Maryam Okhovati, Mohsen Barooni

**Affiliations:** 1 Department of Health Management, Policy and Economics, Faculty of Management and Medical Information Sciences, Kerman University of Medical Sciences, Kerman, Iran; 2 Social Determinants of Health Research Center, Mashhad University of Medical Sciences, Mashhad, Iran; 3 Department of Health Economics and Management Sciences, School of Health, Mashhad University of Medical Sciences, Mashhad, Iran; 4 Department of Library and Medical Information, Faculty of Management and Medical Information Sciences, Kerman University of Medical Sciences, Kerman, Iran; 5 Health Services Management Research Center, Institute for Futures Studies in Health, Kerman University of Medical Sciences, Kerman, Iran

**Keywords:** Resources wastage, Health sector, COVID-19

## Abstract

**Background:**

Identifying factors affecting health costs can contribute to formulating the best policies for controlling and managing health costs. To this end, the present study aimed to analyze resource wastage and identify the factors underlying it in COVID-19 management in Iran’s health sector.

**Method:**

This qualitative content analysis study was conducted in Iran’s health sector. The participants were 23 senior, middle, and operational managers in the health sector. The data were collected through semistructured interviews with the managers. The participants were selected using purposive, quota, and snowball sampling techniques. The interviews continued until the data were saturated. The collected data were analyzed using MAXQDA software (version 10).

**Results:**

Following the data analysis, the factors affecting the wastage of health resources were divided into 4 themes and 13 main themes. Vaccines, diagnostic kits, medicines, and human resources were the main factors underlying resource wastage. The identified main themes were open and unused vials, nonuse of distributed vaccines and their expiration, excess supply and decreased demand for vaccines, expiration of diagnostic and laboratory kits and their quantitative and qualitative defects, and the large number of tests. Inefficiency and the expiration of COVID-19 drugs, poor drug supply and consumption chain management, inaccuracy in inventory control and expiration dates, disorganization and inconsistency in the distribution of healthcare staff, low productivity of the staff, and failure to match the staff’s skills with assigned tasks in selected centers were identified as the most important causes of resource wastage.

**Conclusion:**

Given the limited health funds and the increased healthcare costs, effective preparation and planning and making reasonable decisions for unexpected events can minimize unnecessary costs and resource wastage, which requires some revisions in attitudes toward COVID-19 management in the healthcare sector.

## Background

The sudden outbreak of a new coronavirus in Wuhan Province, China, in December 2019 threatened the world’s population in a short period and caused an increase in the number of COVID-19 cases and deaths in most countries, leading to more than 765 million COVID-19 cases and close to 7 million deaths worldwide by May 2023 [1, 2]. COVID-19 control and treatment have imposed enormous costs on countries both socially and economically. On the one hand, diagnosis, screening, hospitalization, and high treatment costs, and on the other hand, the cost of providing essential health items has put much economic pressure on health systems [3]. Governments have made efforts to manage COVID-19, including immunizing and providing medical equipment (personal protection equipment (PPE), masks, and essential medicines). The total health cost of the response to COVID-19 is estimated to range from USD 234 to 387 billion (accounting for 0.3 to 0.5% of the GDP of the countries of the world) [4].

Iran, with a population of approximately 85 million people [5], a per capita GDP of approximately $4400 [6], and a per capita healthcare expenditure of approximately $570 per year [7], has a mixed health system in which public, private, nongovernmental, and nonprofit sectors are involved in financing, producing resources, and providing medical services [8]. Health and preventive actions are among the important functions of Iran’s health system in dealing with the COVID-19 epidemic. In many countries of the world, the main leading strategy against COVID-19 is prevention and health and is preferred over treatment [9].

With the COVID-19 outbreak in March 2020, the Iranian government decided to allocate one billion euros from the resources of the National Development Fund to address the disease by paying urgent healthcare costs to equip medical centers and provide medicines and medical supplies, providing aid to unemployment insurance funds, and financing knowledge-based companies and domestic production. Thus, 860 million euros were allocated to the Ministry of Health to take action to control COVID-19 [10].

When dealing with COVID-19, Iran was facing problems such as restrictions on diagnostic tests and the lack of COVID-19 vaccines due to international sanctions. Furthermore, the lack of effective monitoring of viral variables and economic sanctions that prevented the supply of medical equipment and medicines restricted the country’s ability to control the coronavirus and maintain public health [11].

Until this date (August 21, 2023), according to statistics from the Ministry of Health and Medical Education, the total number of COVID-19 patients in Iran has reached 7,613,532 cases. Furthermore, 57,123,082 COVID-19 diagnostic tests have been performed in the country. The data also indicated that 7,374,684 patients recovered or were discharged from the hospital, and the number of deaths caused by COVID-19 was 146,321 cases. In addition, to date, 65,238,921 people have received the first dose of the COVID-19 vaccination, and 58,633,579 and 31,733,012 people have received the second and third booster doses, respectively. The total doses of vaccines injected in the country have reached 155,605,512 doses [12].

Since the outbreak of the COVID-19 epidemic, many plans have been put into action in Iran for the early diagnosis, treatment, hospitalization, and recovery of COVID-19 patients, including forming crisis management teams, formulating guidelines and protocols, and screening. One of the problems in COVID-19 management was the lack of adequate evidence and political requirements to control and prevent the spread of the disease. Thus, many plans were put in effect in parallel, and much money was imposed on the government [13]. The draft of the report on the damage caused by COVID-19 on Iran’s economy (Presidential Strategic Studies Center) in December 2020 indicates much damage to the healthcare sector. The damage caused to the health sector in Iran due to the COVID-19 outbreak is estimated at 25 thousand billion Tomans in total ($961.5 M) [14]. According to a study conducted in Iran, the direct costs of COVID-19 for each person have been estimated at 3755 dollars, which is close to the 3045 dollars in the USA. The cumulative estimate of the financial costs imposed by the COVID-19 pandemic in the United States is more than 16 trillion dollars, which is equivalent to approximately 90% of its annual GDP [15, 16]. Approximately 20–40% of the total global health costs are wasted, showing inefficiency in the responsibility and monitoring of the performance of health systems. Thus, adopting efficient, cost-effective, and fair approaches for the community and individuals is necessary. This requires the implementation of healthcare delivery models with a suitable mix to meet people’s health needs [17].

General estimates show that resource wastage in the US healthcare system varies from $600 billion to more than $1.9 trillion per year, or approximately $1,800 to $5,700 per person per year. In 2018, national health expenditures in the United States reached $3.6 trillion, accounting for approximately 18% of GDP. However, this amount of US healthcare spending did little to improve population health outcomes and was mostly wasted. It is estimated that 25% of healthcare spending in the United States is wasted [18].

Cost analysis is a precursor to cost‒benefit analysis, cost-effectiveness, and planning and operational budget formulation. Cost analysis will be useful for managers at different levels to know how the funds were spent, whether these funds have been spent in line with the goals of the organization, and to what extent they have brought the organization closer or further away from its goals [19].

Previous studies have demonstrated some difficulties in determining unnecessary expenses and complex interactions between different causes in the health system. Major changes in resource wastage reduction will not occur as long as different organizations and people in the healthcare system are given clear incentives to reduce resource wastage and the necessary knowledge and tools to perform this task [20].

The COVID-19 epidemic has revealed the inherent value of health and the resilience of the health sector, the importance of adequate investment in health, and the interconnectedness of health and economic processes. Countries that incorporated pandemic preparedness into their health systems were better able to cope with the pandemic and provide access to essential services and continuing care, including key public health functions without financial barriers. The absence of prior planning in an emergency can lead to the wastage of resources and unwanted loss of lives, as well as endangering public trust in medical services [21].

Qualitative research examines events and experiences in a given context with a focus on individual experiences related to that phenomenon [22]. Thus, this qualitative study aims to address resource wastage and identify related factors during the COVID-19 epidemic from the perspective of medical staff. Since no study has addressed resource wastage in Iran’s health system during epidemic conditions, the findings from the present study can provide solutions and suggestions for the effective allocation of resources and preventing their wastage in Iran’s health sector.

## Materials and methods

### Research design

This study was conducted using a qualitative content analysis approach in 2022–2023 in Iran’s health sector.

#### Participants and sampling

The participants in this study were 7 senior health managers (university health deputies), 8 middle managers (staff experts), and 8 executive managers (directors of healthcare centers). The managers were selected using purposive, quota, and snowball sampling techniques. The sampling process continued until the data were saturated. The inclusion criteria were having experience in COVID-19 management for at least one year since the outbreak of the epidemic and the willingness to participate in the study. The exclusion criterion was the unwillingness to continue the interview.

#### Instruments

The data in this study were collected through in-depth and semistructured interviews. The data were documented by taking notes and recording and transcribing the interviews. Each interview started with a general question about the manager’s role and responsibility during the COVID-19 epidemic. More specific questions were asked based on the results of the initial interviews and the categories extracted. For example, “How do managers define resource wastage?” and “In which domains did resource wastage occur, and what are the related factors?”. All interviews were conducted with prior appointments with the participants at their workplace, and in a quiet environment, written informed consent was obtained from the participants before conducting and audio recording the interviews. Each interview lasted ± 33.61 min on average. The interviews were conducted from November 2022 to May 2023.

#### Data analysis

The data collected in this study were analyzed with a conventional content analysis approach using MAXQDA software (version 2020) to extract themes and main themes. Content analysis is an effective method to obtain valid and reliable results from textual data to create new knowledge and insights and provide facts and practical guidance for performance. Content analysis also aims to achieve a brief and exhaustive description of the phenomenon in question [23]. The data were reviewed word by word by two members of the research team. Then, the members of the research team underlined the themes and main themes related to the subject matter in question. The first and last authors had experience in health epidemic management programs, and the third author was experienced in qualitative studies. The data were collected using a semistructured interview guide. The demographic characteristics of the participants were recorded, and the interviews were conducted using a questionnaire. Then, additional probing questions were asked based on the participants’ responses. The interviews were recorded and reviewed again. The audio file of each interview was transcribed word by word. The data were saturated when no new information or theme was discovered with additional interviews. The interviews were reviewed several times by the researcher, and their content was analyzed using the qualitative content analysis approach proposed by Graneheim and Lundman [24]. Afterward, the meaning units were identified and coded. In the next step, the codes were clustered into main themes based on semantic similarities and differences. Moreover, the main themes with conceptual and semantic connections were organized into more abstract categories. Finally, the main themes were extracted by merging the themes that covered a single concept [25]. The data were saturated with 23 semistructured interviews. To establish the rigor of the study, the four criteria of trustworthiness, credibility, dependability, confirmability, and transferability proposed by Lincoln and Guba were used [26]. The credibility of the findings was established through the prolonged engagement of researchers in different stages of the study, reviewing the content of each interview, reading the interview transcripts several times, presenting the transcripts to the participants, and revising them based on their feedback. Finally, the extracted codes and categories and the conceptual model were reviewed by a panel of experts and revised based on the feedback received from them. The findings were reviewed and confirmed according to the participants’ opinions. However, to further ensure the confirmability of the findings, they were reviewed by an expert experienced in qualitative methods. The dependability of the data was enhanced by recording all procedures taken to conduct the study and conducting all interviews under similar conditions. To ensure the transferability of the findings, the researchers were constantly engaged in all research procedures.

## Results

Using qualitative content analysis, this study examined the factors underlying the wastage of health resources. Interviews were conducted with 23 health deputies, managers, and experts. Most of the participants (78%) were male. The participants’ average age was ± 47.8 years, their average work experience was ± 21.96 years, and their average managerial experience was ± 14.7 years in the health sector.

Table 1 shows other demographic data of the participants. After removing overlaps, 345 codes were extracted from 614 initial codes. Data analysis revealed 4 themes (COVID-19 vaccines, diagnostic kits, medicines and medical supplies, and human resources) and 13 main themes, as shown in Table 2.

**Table 1:** Demographic characteristics of the interviewees

Interviewee	Years of service	Management experience	Age	Gender	Field of study	Education Level	Interview Duration (min)
P1	11	6	38	Female	Cardiology	Ph.D.	40
P2	20	11	44	Male	Biochemistry	Ph.D.	42
P3	35	33	58	Male	Psychology	Ph.D.	33
P4	18	14	43	Male	Health Economics	Ph.D.	42
P5	19	12	50	Female	General Practitioner	Professional Doctorate	30
P6	18	13	43	Female	General Practitioner	Professional Doctorate	38
P7	22	10	50	Male	General Practitioner	Professional Doctorate	50
P8	15	4	43	Male	General Practitioner	Professional Doctorate	39
P9	25	21	52	Male	General Practitioner	Professional Doctorate	30
P10	28	15	61	Male	General Practitioner	Professional Doctorate	35
P11	24	20	52	Male	General Practitioner	Professional Doctorate	28
P12	25	28	58	Male	Pharmacist	Professional Doctorate	35
P13	22	25	53	Male	Pharmacist	Professional Doctorate	25
P14	31	21	57	Male	Laboratory Sciences	Master’s Degree	28
P15	20	11	42	Male	Laboratory Sciences	Master’s Degree	48
P16	23	12	49	Female	Psychology	Master’s Degree	25
P17	30	18	52	Female	Laboratory Sciences	Bachelor’s Degree	27
P18	18	7	41	Male	Laboratory Sciences	Bachelor’s Degree	34
P19	15	4	36	Male	Public Health	Bachelor’s Degree	28
P20	29	21	53	Male	Public Health	Bachelor’s Degree	27
P21	20	11	43	Male	Public Health	Bachelor’s Degree	25
P22	19	8	45	Male	Public Health	Bachelor’s Degree	34
P23	18	13	37	Male	Pharmaceutical Affairs	Bachelor’s Degree	30

**Table 2:** The themes, main themes, and subcategories

Themes	Main Themes	Subcategories
COVID-19 vaccination	Open or unused COVID-19 vials	The high doses contained in each vaccination vial
		Unsuitable COVID-19 vaccination storage conditions
		Human error in the vaccination process
	The nonuse of COVID-19 vaccines distributed among the centers and the expiry of vaccines	Changes in the need and demand for vaccines
		People’s unwillingness to do vaccination
		Public mistrust in existing COVID-19 vaccines
	Mismanagement of ***the*** distribution and redistribution of COVID-19 vaccines	Improper distribution of COVID-19 vaccines in vaccination centers
		Failure to redistribute near-expired COVID-19 vaccines
	Excess supply and decreased demand for the COVID-19 vaccines	Diverse or ineffective planning for producing COVID-19 vaccines inside the country
		Multiplicity and variety of vaccination programs and plans
COVID-19 diagnostic and laboratory kits	Expiration of COVID-19 diagnostic and laboratory kits	A reduction in the number of people referring for COVID-19 screening and diagnostic tests
		Excessive provision of COVID-19 diagnostic and laboratory kits without assessing the epidemic conditions
		Failure to redistribute near-expired COVID-19 diagnostic kits
	Quantitative and qualitative defects in COVID-19 diagnostic kits	Noncompliance with the COVID-19 kits sent from the ministry with the laboratory equipment in medical center
		Unavailability of valid COVID-19 diagnostic and laboratory kits in the market
		Errors in COVID-19 diagnostic and laboratory tests and the need to repeat the tests
	A large number of COVID-19 tests	Conducting routine and induction COVID-19 diagnostic tests
		Conducting diagnostic tests to prepare statistics and report the COVID-19 trends
		Applying for PCR tests to confirm COVID-19
COVID-19 medicines and medical supplies	Ineffectiveness and expiration of COVID-19 medicines	Frequent changes in drug treatment protocols for COVID-19
		Changes in treatment algorithms due to medical advances
	Poor drug supply and consumption chain management	Indiscriminate preparation and distribution of medicines without assessing the COVID-19 condition
		Not redistributing near-expired COVID-19 drugs
	Inaccurate inventory drug and expiry date control	Inaccurate assessment of needs and reasonable consumption
		Absence of a regular inventory and consumption recording and tracking system
Human resources	Disorganization and inconsistency in distributing staff in medical centers	Making decisions and formulating instructions based on the function of selected COVID-19 centers
		Ineffective planning for staff distribution
		Ineffective use of human resources
	Low productivity of the staff in selected COVID-19 centers	Nonoptimal use of staff capacities
		Lack of adequate experience and skills in medical staff in selected COVID-19 treatment and vaccination centers and reference laboratories
		Reduced staff productivity and absenteeism due to COVID-19
	The mismatch between staff’s skills and assigned tasks in medical centers	Lack of updated job descriptions and mismatch with the staff’s expectations

The COVID-19 epidemic is managed differently in countries depending on local and institutional conditions. The factors underlying resource wastage include ineffective planning, lack of preparation, the absence of a crisis management plan, lack of communication and coordination, and lack of coordination between different management levels. The findings from the present study showed that the factors underlying resource wastage include COVID-19 vaccination, diagnostic and laboratory kits, medicines and medical supplies, and human resources, as discussed below:

## COVID-19 vaccination

The current strategy for addressing the COVID-19 pandemic is the optimal use of vaccines to reduce COVID-19 cases and mortality [27]. An analysis of the participants’ experiences showed that factors such as open and unused vials, nonuse of distributed vaccines, excess vaccine supply, and reduced demand contributed to vaccine waste.

### Open or unused COVID-19 vials

Unopened vials are wasted mostly due to cold chain, supply management, and storage problems. The participants stated that the amount and number of doses in each vial and vaccine can be the main reason for the wastage of COVID-19 vaccines.

#### High doses in each COVID-19 vaccine vial

Wastage in vaccines is often due to the characteristics of the vial itself and the number of doses. According to the participants, this problem can be caused by the differences in the doses in different vials. A participant stated:

“*The doses in vaccine vials put us in a lot of trouble. We were not sure whether to open the vial or not*” (Participant #20).

#### Unsuitable COVID-19 vaccination storage conditions

Unsuitable storage of vaccines reduces their effectiveness or leads to their spoilage. Most of the participants stated that not keeping vaccines at the optimal temperature in the vaccination process can contribute to the wastage of vaccines. A participant stated:

“*The storage temperature of the vaccines inside the vaccination cold box was the same as the ambient temperature in some cases*” (Participant #22).

#### Human error in the vaccination process

Human errors can occur in the vaccination process and cause some problems. Some participants pointed out that human errors also contributed to the waste of vaccines. A participant reported:

“*The vaccinator used to forget to place open and unused vials in the refrigerator and cold box and noticed the problem just the next day*” (Participant #20).

### The nonuse of vaccines distributed among centers and expiration of COVID-19 vaccines

The waste of COVID-19 vaccines due to expiration indicates defects in vaccine management and planning. The participants stated that the lack of referral and demand for vaccines, doubt and unwillingness of community members, and mistrust in vaccines available in the vaccination program could lead to the wastage of COVID-19 vaccines.

#### Changes in the need and demand for vaccines

The successful implementation of the COVID-19 vaccination program, in addition to the efficiency and effectiveness of the vaccines, will require public acceptance and the cooperation of all members of the community to fight this disease. Most of the participants emphasized that one of the problems with the vaccination program was the lack of referrals for vaccination. A participant stated:

“*People don’t like to visit our vaccination centers as if COVID-19 is over for them*” (Participant #19).

#### People’s unwillingness to undergo vaccination

Hesitation and delay in refusing vaccination are obstacles to long-term control of the coronavirus. The participants stated that reasons such as negative publicity, allocation of vaccines to specific groups, complications, and ambiguities about the consequences and effects of the vaccines could contribute to the wastage of vaccines. A participant stated:

“*Fear of vaccines and the negative publicity and beliefs of some groups such as traditional medicine professionals and clerics were also effective in reducing referrals and wastage of vaccines*” (Participant #7).

#### Public mistrust in the existing COVID-19 vaccines

The participants stated that various factors may contribute to public mistrust in COVID-19 vaccines, including confusion caused by an enormous bulk of information and the association between vaccination and side effects and certain diseases. One participant said:

“*We politicized vaccination and even its production turned into a political issue. They produced different types of COVID-19 vaccination, and none of them were effective, and people became skeptical of the vaccines*” (Participant #3).

### Mismanagement of the distribution and redistribution of COVID-19 vaccines

Effective management in the distribution of vaccines is important in infectious crises and requires effective planning, careful monitoring of the vaccine supply chain, equitable distribution, and increasing the capacity of health infrastructure.

#### Improper distribution of COVID-19 vaccines in vaccination centers

The participants reported that the ineffective distribution of health resources and services leads to their wastage and inefficient use of them. Most of the participants stated the ineffective distribution of vaccines, the reduction of visits to vaccination centers, and the failure to redistribute near-expired vaccines as reasons for the wastage of vaccines. A participant stated:

“*Sometimes we were receiving vaccines that we had no choice but to accept. It appeared to be they were forcing us to take those vaccines regardless of the demand for vaccination*” (Participant #22).

#### Failure to redistribute near-expired vaccines

A majority of the participants agreed that adopting a collaborative, redistributive, or hybrid approach to an existing vaccination program would increase vaccine access and reduce vaccination wastage. One of the participants stated:

“*We told the vaccination centers in different provinces that we were going to send them some near-expired vaccines, but they replied that they had lots of unused vaccines and could send them back*” (Participant #4).

### Excess supply and decreased demand for vaccines

Vaccine oversupply means more production than demand. This problem may be due to overproduction or distribution problems. The participants stated that diverse or ineffective planning for producing COVID-19 vaccines and the multiplicity of vaccination programs were the reasons for the wastage of vaccines .

#### Diverse or ineffective planning for producing COVID-19 vaccines inside the country

The production of COVID-19 vaccines is a priority for countries as a preventive measure to control the spread of the disease. The participants in this study expressed concerns such as high production costs, insufficient time for clinical trials, the lack of international scientific certificates for domestic vaccines, and the lack of community participation in vaccination acceptance. One of the participants stated:

“*In a scenario where the coronavirus was rampant in Iran and hundreds of people succumbed to the virus every day, priority was given to domestic vaccine production over the timely import of foreign vaccines*” (Participant #4).

#### Multiplicity and variety of vaccination programs and plans

The successful fulfillment of vaccination goals requires effective and integrated planning in implementing the vaccination project. A participant stated:

“*Mobile vaccination plans were carried out in certain places such as Friday prayers, holidays, gatherings, crowded markets, and offices, but people attended these places for nonvaccination reasons, which caused a large number of vaccines to be wasted*” (Participant #19).

## COVID-19 diagnostic and laboratory kits

The use of suitable laboratory equipment plays an essential role in confirming and diagnosing diseases on time. The disregard for such equipment can lead to delays in the treatment and timely diagnosis of diseases [28]. From the perspective of the participants, factors such as the expiration date of the kits, the quality of equipment and their brand, and the provision of free diagnostic tests played an important role in the wastage of the kits.

### Expiration of COVID-19 diagnostic and laboratory kits

The participants in the study stated that a reduction in the number of patients referred for vacation due to the slower COVID-19 transmission rate, excessive supply of diagnostic kits without evaluating the disease trend, dependence on the past behavior of the coronavirus, and failure to redistribute additional kits were effective in the expiry of COVID-19 diagnostic and laboratory kits.

#### A reduction in the number of people referred for COVID-19 screening and diagnostic tests

The participants stated that a reason for the decrease in the number of COVID-19 tests was an increase in public awareness and health literacy. One of the participants stated:

“*No one applied for COVID-19 screening and diagnostic tests, and the officials asked why the number of diagnostic tests had reduced, but they knew that there was a decrease in the number of COVID-19 patients across the country*” (Participant #18).

#### Excessive supply of COVID-19 diagnostic and laboratory kits without evaluating the Disease trend

Most of the participants emphasized that COVID-19 diagnostic and laboratory kits and other medical equipment should be supplied according to a detailed analysis of the different COVID-19 waves in the country, and decisions should not be made based on the past behavior of the coronavirus or improper preparation and distribution of laboratory kits. A participant with a surprised and angry expression stated:

“*If the university was appointed as the main authority in charge of supplying COVID-19 kits from the first day, or if the requested kits were delivered to the university based on the submitted data, we would not have so many extra or near-expired kits*” (Participant #14).

#### Failure to redistribute near-expired COVID-19 diagnostic kits

The participants stated that with effective planning, the authorities could monitor the received and purchased kits and redistribute the near-expired kits, thus preventing the purchase of additional kits. One of the participants said:

“*The authorities bought or sent as many diagnostic kits as they could. They did not think what should be done if the COVID-19 epidemic subsided one day. No one thought of redistributing them among other universities and laboratory centers to at least prevent buying and wasting additional kits*” (Participant #4).

### Quantitative and qualitative defects in COVID-19 diagnostic tests

Higher-quality diagnostic and laboratory kits produce more accurate and reliable results. The participants reported some problems, such as the incompatibility of kits with laboratory devices, the availability of different brands of kits on the market, and recurrent errors that could be problematic in conducting experiments.

#### Noncompliance of the COVID-19 kits sent from the ministry with the laboratory equipment in medical centers

Diagnostic and laboratory kits are very important for diagnosing diseases. A participant stated, “*Diagnostic kits were of different models and the devices were more or less sensitive to some kits*” (Participant #17).

#### Unavailability of valid COVID-19 diagnostic and laboratory kits

Some of the participants stated that the accuracy and reliability of diagnostic kits are very important for the correct diagnosis of suspected and confirmed COVID-19 cases, and any defective diagnosis can have serious effects on people’s health. A participant stated:

“*When the PCR sample extraction kits made abroad were replaced with kits produced in the country, we had many troubles. We had strange problems with these kits and all kinds of domestic brands*” (Participant #2).

#### Errors in COVID-19 diagnostic and laboratory tests and the need to repeat the tests

The participants stated that several factors may affect the test results, producing unreliable results. A participant stated:

“*There are many more errors in rapid tests than PCR tests. We got into a lot of trouble*” (Participant #18).

### A large number of COVID-19 tests

Most of the participants confirmed a large number of COVID-19 tests due to the abundant use of diagnostic and laboratory kits. They confirmed that the unreasonable performance of PCR tests by hospitals, diagnostic tests to report the statistics of patients and monitor the course of the disease, and the confirmation of sick leave by medical staff added many costs to the health sector. A participant stated:

“*Everyone expected that they would be given a PCR test It had become a common practice for clients. Maybe their fear of the disease had prompted them to do this*” (Participant #4).

#### Conducting routine and induction COVID-19 tests

Most of the participants admitted that many of the samples sent from the hospital to diagnose the disease had nothing to do with the symptoms and disease of the person who was going to be hospitalized. A participant stated, “*Even if the patient was a small child and needed to be admitted to the hospital, samples were still taken from them. They truly did not have any of the symptoms of COVID-19, and there was no need for such tests*” (Participant #7).

#### Conducting diagnostic tests to prepare statistics and report the COVID-19 trend

Following the instructions of the Ministry of Health, positive COVID-19 cases in the country were detected and confirmed only through PCR tests for suspected and infected patients. However, many participants believed that in severe COVID-19 peaks with a large number of positive cases, it was not necessary to carry out PCR testing on a large scale. A participant stated:

“*PCR tests are administered for surveillance and monitoring purposes. However, PCR testing was of no value during COVID-19 peaks because the screening of the disease was no longer an issue as it was at the beginning of the disease*” (Participant #5).

#### Applying PCR tests to confirm COVID-19

Iran’s Ministry of Health has adhered to evidence-based policymaking since the COVID-19 outbreak. However, there were many inconsistencies between different departments in implementing guidelines and recommendations. Most of the participants stated the problems of conducting medical tests for employees due to inconsistency between public and private organizations. A participant said:

“*We had a client who was very angry and had a military job. He insisted that the PCR test be done for him. His organizational manager only confirmed his COVID-19 leave based on a positive PCR test. So we had to do it*” (Participant #10).

## COVID-19 medicines and medical supplies

Constant changes in treatment protocols and defects in the distribution, purchase, and storage of drugs, along with the lack of strict supervision and control, have led to the waste of a significant amount of medicines in healthcare centers [29]. The majority of the participants stated that most of the medicines available in these centers remained in the warehouses due to their inefficiency or expiry.

### Ineffectiveness and expiration of COVID-19 medicines

Expired medications may result in reduced effectiveness and potency. Most of the participants confirmed that factors such as changing treatment protocols, poor supply management, consumption of drugs, inaccurate inventory control, and expiration date played an important role in the wastage of COVID-19 medicines.

#### Frequent changes in drug treatment protocols for COVID-19

The participants stated that during the COVID-19 epidemic, prescribed drugs for COVID-19 patients were changed in Iran due to controversies among medical professionals and experts, and there are still no uniform guidelines for the introduction of effective drugs for the COVID-19 epidemic. A participant stated:

“*At the onset of the COVID-19 epidemic, hydroxyl chloroquine was one of the drugs prescribed to patients, but later, it was proven that this drug has nothing to do with the treatment of this disease*” (Participant #11).

#### Changes in treatment algorithms with the advancement of medical knowledge

Most of the participants stated that the changes in treatment algorithms were due to improved medical procedures, more knowledge about diseases, the discovery of new medicines, and changes in standards leading to some drugs becoming outdated and ineffective. A participant stated:

“*With the progress of the disease, new drugs were introduced and used in the treatment cycle, and the effectiveness of other drugs that were used before was questioned*” (Participant #1).

### Poor drug supply chain and consumption chain management

Poor supply chains and consumption management can lead to the early expiration and ineffectiveness of COVID-19 medicines. Most of the participants stated that the unreasonable procurement and distribution of drugs and failure to redistribute near-expired COVID-19 medicines were mainly caused by poor supply chains and consumption management of COVID-19 medicines.

#### Indiscriminate preparation and distribution of medicines without assessing the COVID-19 condition

The participants reported that excessive loads of COVID-19 medicine were sent to medical and healthcare centers by the Infectious Disease Management Center of the Ministry of Health or the university. One of the participants stated:

“*COVID-19 medicines were sent to us by the Ministry of Health. We did not order these medicines. This trend continued even until recently and the decline of the disease*” (Participant #23).

#### Not redistributing near-expired COVID-19 Drugs

The majority of the participants stated that some of the COVID-19 drugs that were not used gradually expired, were collected and were destroyed. The redistribution of drugs by the Ministry of Health and the university could keep these drugs in the cycle of production and reuse. A participant said:

“The officials of the Ministry of Health and the University of Medical Sciences could not make a proper decision about the remaining COVID-19 drugs. Thus, many of these drugs remained in the warehouses of healthcare centers and were wasted” (Participant #12).

### Inaccurate inventory drug and expiry date control

Inaccurate inventory control and expiration date of COVID-19 drugs can lead to the use of expired drugs, wastage of drugs, and financial losses. Most of the participants highlighted the importance of factors such as inaccurate assessment of needs and reasonable consumption, the absence of a regular registration and tracking system, and ineffective inventory and consumption control.

#### Inaccurate assessment of needs and reasonable consumption

Most of the participants reported that the inaccurate assessment of the needs for medicines and their reasonable consumption in each sector may lead to a mismatch with the needs of patients, resource wastage, and decreased efficiency of medicines. One participant stated:

“Without an accurate assessment of needs and reasonable consumption in each sector, we actually don’t have enough drugs or use drugs ineffectively” (Participant #12).

#### Absence of a regular inventory and consumption recording and tracking system

Most of the participants complained about the absence of a regular inventory and consumption recording and tracking system and stated that it can lead to the wastage of drugs, inconsistency in the ordering and supply of drugs, loss of resources, and less efficient use of drugs. A participant stated:

“*The absence of a robust system to regularly record and track drug inventory and consumption caused some important COVID-19 drugs to expire suddenly*” (Participant #13).

## Human resources

Controlling human resource costs is one approach that has a significant impact on the performance of organizations. Neglecting this issue can have negative consequences and affect the quality of service delivery by the organization [30]. The participants stated that the lack of organization and planning for the effective distribution of medical staff in screening and diagnostic centers can play an important role in increasing costs. The participants also suggested that the lack of organization and inconsistency in the distribution of staff, low efficiency of the staff, and problems in assigning tasks can play an important role in increasing human resource costs.

### Disorganization and inconsistency in distributing staff in medical centers

Organization and coordination in the distribution of medical staff are two factors that affect the efficient performance and success of the organization. The participants stated that the decisions and instructions related to the operation of the centers, the lack of effective planning for the distribution of the staff, and inefficient use of the staff have led to the waste of costs in the payment of salaries and benefits.

#### Making decisions and formulating instructions based on the function of selected COVID-19 vaccination centers

To function effectively in dynamic environments, assessing the current situation is essential. Participants repeatedly acknowledged that decisions were typically made differently during crises due to time and documentation constraints. A participant stated:

“*The number of visitors and the number of vaccinations were not proportionate to the number of medical staff working in vaccination centers. A few months earlier, the authorities should have closed some vaccination centers to avoid wasting funds and resources*” (Participant #7).

#### Ineffective planning for staff distribution

A majority of the participants reported that inefficient planning of staff distribution led to the wastage of human resources and time. A participant stated:

“*Some medical centers had excessive staff, while some had a shortage of staff, including sampling technicians and nurses*” (Participant #4).

#### Ineffective use of human resources

The majority of participants stated that during the COVID-19 pandemic, ineffective use of medical staff and inefficient assignment of tasks increased costs. For instance, a participant stated:

“*The employment of volunteers in healthcare centers was one of the problems. On one hand, their admission could disrupt healthcare services, and on the other hand, their unemployment could demotivate them*” (Participant #6).

### Low productivity of the staff in selected COVID-19 centers

Efforts to improve and effectively use various resources, such as labor, capital, materials, energy, and information, are considered important in organizations. Most of the participants stated that inefficient use of the existing medical staff, hiring inexperienced and unskilled staff in the centers, reduced productivity, and absenteeism led to resource wastage during the COVID-19 pandemic.

#### Nonoptimal use of staff capacities

The participants suggested that nonoptimal use of staff capacities caused an increase in the costs and salaries of the staff. Furthermore, the Ministry of Health was forced to hire contractual medical staff to control the COVID-19 pandemic. Perhaps the ministry did not need to do so. A participant stated:

“*Some medical centers employed contractual staff for only 86 days. These staff were redundant. Even some of our staff had nothing to do at all*” (Participant #4).

#### Lack of adequate experience and skills in medical staff in selected COVID-19 treatment and vaccination centers and reference laboratories

According to some participants, factors such as inefficient selection and recruitment processes, low knowledge, competencies, and skills of medical staff, and lack of any preparation for the staff were key challenges in addressing COVID-19, leading to the waste of resources and funds. A participant stated:

“*Some of the medical staff in reference laboratories and selected vaccination centers did not have any work experience and were employed in these centers immediately after starting their human resource plan, which was a mistake*” (Participant #9).

#### Reduced staff productivity and absenteeism due to COVID-19

Most of the participants stated that contracting COVID-19 had reduced the productivity of staff and led to absenteeism. Reduced productivity, the need for alternative workforces, and the destruction of human capital were the factors that contributed to increasing the wastage of funds and resources. A participant stated:

“*In the first month, almost half of the staff in medical and screening centers were infected with COVID-19 and took sick leave. Unfortunately, we had to get help from the staff in other departments and centers*” (Participant #8).

### the mismatch between staff skills and assigned tasks in medical centers

To reduce the mismatch between staff skills and assigned tasks, we should pay special attention to the appropriate design of tasks, upgrading skills through training and development, and creating an organizational culture that promotes the skills and abilities of staff. A majority of the participants in this study stated that the staff’s job description did not match their expectations, leading to the waste of resources.

#### Lack of updated job descriptions and mismatch with the staff’s expectations

Most of the participants stated that updated job descriptions may lead to a lack of clarity in tasks, inability to perform tasks, inefficiency in resource allocation, waste of time and energy, and nonpromotion of capabilities, which are rooted in the structure of the organization. A participant stated:

“*At the outbreak of COVID-19, everyone was confused, there was little training, and no one knew what to do*” (Participant #2).

## Discussion

Given the limited financial resources in the healthcare system and increased healthcare costs, reasonable measures and planning for emergencies can help to minimize resource wastage and unnecessary costs and manage resources more efficiently. The present study aimed to identify the most important cost centers and related factors during the COVID-19 pandemic in the health sector in Iran. Four factors leading to resource wastage were vaccines, diagnostic and laboratory kits, medicines and medical supplies, and workforce.

The first factor leading to resource wastage was COVID-19 vaccines. The previous studies that have addressed the wastage of vaccines have suggested that factors such as the characteristics of vaccination vials, public mistrust in existing COVID-19 vaccines, dissemination of false information about vaccines, the existence of vaccine-resistant groups, rumors surrounding them, expiration of vaccines, inefficient distribution and delivery of vaccines, and failure to redistribute them to provider centers affected the distribution of COVID-19 vaccines [31, 32]. In addition, our study reported that excess supply and demand and the multiplicity of COVID-19 vaccines led to vaccine wastage. It is estimated that more than one billion doses of the COVID-19 vaccine have been wasted, 8% of which are vaccines that have remained unused. For example, in Georgia, out of a total of 5,334,060 doses, 408,801 thousand doses of different types of vaccines, such as AstraZeneca, Sinopharm, and Sinvac, were wasted mainly due to public reluctance and low demand for vaccines, as confirmed in the present study [33, 34]. In Canada, since the start of COVID-19 vaccination, approximately one million doses of vaccines have been wasted, with 12% of the vaccines being expired [35], as highlighted by our study. According to the WHO guidelines, the maximum amount of waste in vaccines is set between 15 and 25%, and in some vaccines (with 2 or 5 doses), it should not exceed 5% [36]. The findings from the present study suggested that to reduce wastage in vaccines, there is a need to develop detailed and organized plans and processes. Suggested strategies can include identifying the wastage rate and its root causes, efficient management of vaccine products, and strengthening the supply chain management system. The engagement of healthcare organizations and medical centers can help ensure the transparent and traceable distribution of vaccines. Vaccination plans and programs must also be put into practice through a robust management system. Innovative communication strategies can be used to increase the acceptance and use of vaccines in the community. These strategies should focus on factors underlying vaccine hesitancy to increase public confidence in vaccines.

The second theme highlighted by the participants in the present study was the waste of diagnostic and laboratory kits. The importance of accessing correct and valid laboratory results has always been one of the goals of laboratory systems. However, laboratory services, despite their essential role in the diagnosis and management of many pathologies, are usually considered one of the main cost centers of the health sector [37]. The data in our study indicated that one of the causes of the wastage of diagnostic and laboratory kits was the conduct of countless tests at COVID-19 peaks in Iran. Various studies have shown that COVID-19 diagnostic tests are undoubtedly useful epidemiological tools, especially for estimating the prevalence of asymptomatic COVID-19 cases. If it is truly the case that only 5% or more of the population is infected with SARS-CoV-2, performing millions of antibody tests may do little to clarify the overall prevalence picture and may not be necessary [38]. The present study also demonstrated that the wastage of diagnostic and laboratory kits can be attributed to secondary factors such as excessive production of COVID-19 diagnostic kits and the failure to redistribute near-expired kits. Additional treatments and low-value tests and care in health systems due to lack of scientific verification or efficiency can significantly contribute to wasting costs and resources. William et al. examined resource wastage in the US health system and noted that additional treatments and low-value care led to unnecessary screening and testing in 2019, imposing 75 to 101 billion dollars on the health system [39]. Similarly, the present study showed that at the COVID-19 peaks, most of the clients who had clinical symptoms of the disease insisted on performing unnecessary diagnostic tests based on the instructions and decisions of the COVID-19 control center, leading to low-value and repetitive additional medical services. If the diagnosis criterion was based on clinical COVID-19 symptoms, a lower number of tests was needed. Thus, considering the importance of wastage in diagnostic and laboratory kits in acute and unusual conditions that are urgently needed, planning to improve laboratory services according to the disease conditions, peaks, and genetic mutations of the virus, predicting the number of kits and laboratory items needed, the selection of suitable and reliable equipment and kits, supply chain management (estimation, planning, distribution, and monitoring), and the use of qualified staff can contribute to improving and promoting diagnostic and laboratory services, as well as reducing the wastage of resources spent on performing diagnostic and laboratory kits. The data in the present study indicated that the third factor leading to the wastage of medicines and medical supplies was the nonuse of drugs and their expiration in healthcare centers, leading to the wastage of valuable resources. Optimal planning and management of the inventory of medicines and medical supplies can contribute to preventing resource wastage and improving the efficiency of the health system.

To better supply medicines and medical supplies in medical centers, the correct functioning and safety of medicines, as well as their distribution and supply processes, are very important. Accurate and efficient management of the supply, storage, and distribution system of medicines is one of the most essential effective factors in guaranteeing the quality of healthcare services. Accordingly, the participants in the present study emphasized the importance of this issue. Medicines play an important role in the relationship between the patient and health services. Inefficient management of medicines, especially in the public sectors of developing countries, is an important challenge, the improvement of which can lead to cost savings and increased public access to medicines [40]. A study conducted from 2012 to 2014 in the Federal City of Addis Ababa showed that the average rate of drug wastage during this period was 4.8%. This waste comes from weak management, lack of audit procedures, transparency, and accountability in the Ethiopian pharmaceutical system. These problems can increase healthcare costs and disrupt the improvement and optimization of the pharmaceutical system [41]. Likewise, the present study also indicated that factors such as inaccurate assessment of needs and reasonable consumption and the absence of a regular inventory and consumption recording and tracking system contributed to the wastage of drugs. Furthermore, Abera Bedasa Alemu et al. in 2023 showed that the wastage of medicines in Ethiopia is due to the delivery of near-expired medicines to medical centers, sudden changes in treatment protocols, and overstocking of medicines due to the inaccurate prediction of drug requests during the COVID-19 epidemic. Inadequate inventory control is responsible for 4–9% of drugs being wasted in supply systems. More than half of drugs are improperly prescribed, distributed, or sold, especially in developing countries, where there are limited regulatory procedures for the use of drugs. Improving accurate inventory management and effective regulatory measures can prevent the wastage of drugs and valuable resources [42]. According to the present study and other studies in the literature, there is currently no specific, effective, and proven treatment available for COVID-19. Different drugs have different effects on patients with COVID-19, and there is a need for more investigations on the potential usefulness or harmfulness of the drugs proposed for the treatment of this disease [43].

Roustit et al. examined hydroxychloroquine and its effect on COVID-19 in laboratory conditions and confirmed its effectiveness against the SARS-CoV-2 virus. However, as of mid-2020, clinical data have failed to confirm the effectiveness of these drugs in patients with COVID-19. On the other hand, the unscientific advertising of this drug led to the execution of more than a hundred studies and endangered resources and delays in more reliable tests for COVID-19 treatment [44]. Similarly, the present study indicated that the drugs are ineffective due to frequent changes in medicinal protocols for treating COVID-19. Since the waste of medicines imposes heavy costs on public health and the economy, innovative solutions, including the use of advanced technologies to improve the shelf life of drugs, more accurate methods for managing drugs, optimizing drug consumption, and advanced drug information and management systems for more accurate tracking of drug inventory, are recommended to improve drug management and reduce health and economic costs.

The fourth factor identified in this study was the wastage of human resources in the management of the COVID-19 epidemic. One of the key elements of the health system in the prevention, control, and treatment of COVID-19 can be healthcare and medical staff. Currently, the effects of socioeconomic, technological, and cultural changes lead to continuous complexity and dynamics in health systems. One of the key factors in continuous success and achieving goals in these complex environments is human resources [45]. The lack of professional staff, low knowledge of staff, and ineffective distribution of their skills can be a major obstacle to achieving organizational development goals [46, 47]. Carnevale et al. showed that the success of organizations depends on an optimal mix of financial, human, and logistical resources to achieve short-term and long-term goals [48].

Tabatabaei et al. examined lost productivity caused by absenteeism due to the COVID-19 epidemic in Iran and showed that the total costs due to the absence of 1958 staff were nearly 1.3 million dollars, with an average of 671.4 dollars for each of the sick staff, indicating a significant economic burden and confirming the importance of this issue in resource wastage in the COVID-19 crisis [49], as reported in the present study. Other studies have also addressed human resource management challenges during the COVID-19 pandemic. The data in the present study showed that the lack of effective planning and proper utilization of human resources, nonoptimal use of the available capacities of the staff, and the lack of effective evaluation of the staff performance and necessary training contributed to wasting costs, caused problems related to human resources, and imposed costs on the organization, as confirmed in other studies that have addressed organizational challenges from the perspective of human resources [50]. Although healthcare systems around the world are increasingly facing challenges in human resources and improper distribution of their skills, applying optimal management along with other practical plans to improve the quality of these resources can lead to the improvement of competencies, the quality of service delivery, and the reduction of related challenges. The organization and coordination of human resources and the optimal use of existing capacities require more detailed planning and policies to prevent the wastage of financial resources, time, and other costs.

## Limitations of the study

One of the limitations of this study was the limited generalizability of its findings. However, the instructions and protocols provided by the Ministry of Health to control the COVID-19 epidemic in the health sector are similar and identical. Thus, the findings of this study can be generalized with caution. Another limitation of the study was the unavailability of experts and policymakers at the level of the Ministry of Health and Medical Education and deputies and academic administrators because of managerial changes in the new government of Iran. Moreover, given the sensitivity of the subjects and the wastage of some resources such as vaccines, some participants were hesitant to attend the interviews for political reasons.

## Conclusion

Healthcare systems are struggling with an unprecedented crisis. The crisis caused by an infectious pandemic has revealed a very different dimension of management in the health systems of countries. The COVID-19 epidemic and other crises present us with opportunities to accelerate some of the changes we may already have or to challenge some long-held assumptions and think more creatively about new procedures. Given the limited health funds and the increased healthcare costs, effective preparation and planning and making reasonable decisions for unexpected events can minimize unnecessary costs and resource wastage, which requires some revisions in attitudes toward COVID-19 management in the healthcare sector. Cost management is based on the view that costs do not arise by themselves, but management decisions involve how to use the organization’s limited resources. Accordingly, solutions such as effective planning, strengthening of health infrastructure, formulation and implementation of protocols and detailed instructions, promotion of public awareness, cooperation and coordination between institutions, review and evaluation of current costs, review and optimization of work processes, reduction of labor costs, improvement of inventory management, accurate planning, correct recognition of needs, and use of innovative solutions to reduce costs and maintain service quality, trying to reduce costs of procurement of medicines, equipment, and consumables, and optimal use of available resources can minimize the wastage of resources. To determine what is “routine”, what is “necessary” and what is “exceptional”, it is necessary to make medical and health decisions in the paradigm of high-quality and effective care, with minimal resource waste, and to adopt the best healthcare solution and initiatives in acute and critical conditions. By taking preventive measures in potential scenarios and having effective plans, health systems can ensure the efficient allocation of resources. In addition, any unexpected event can be addressed quickly and efficiently. Such measures can contribute to minimizing costs and wasting resources, especially in times of bad economic conditions or when resources are scarce. Thus, it is essential to have a proactive approach to crisis management that is both cost-effective and efficient.

## Data Availability

The datasets generated and analyzed during the current study are not publicly available due to confidentiality concerns and institutional regulations. However, reasonable requests for data access may be considered by contacting the corresponding author.
